# Gravel Bars Can Be Critical for Biodiversity Conservation: A Case Study on Scaly-Sided Merganser in South China

**DOI:** 10.1371/journal.pone.0127387

**Published:** 2015-05-21

**Authors:** Qing Zeng, Linlu Shi, Li Wen, Junzhu Chen, Hairui Duo, Guangchun Lei

**Affiliations:** 1 School of Nature Conservation, Beijing Forestry University, Beijing, 100083, China; 2 Water Wetlands and Coasts Science, NSW Office of Environment and Heritage, Sydney, NSW, 2001, Australia; Institut Pluridisciplinaire Hubert Curien, FRANCE

## Abstract

Gravel bars are characteristic components of river landscapes and are increasingly recognized as key sites for many waterbirds, though detailed studies on the ecological function of gravel bars for waterbirds are rare. In this study, we surveyed the endangered Scaly-sided Merganser *Mergus squamatus* along a 40 km river section of Yuan River, in Central China, for three consecutive winters. We derived the landscape metrics of river gravel bars from geo-rectified fine resolution (0.6 m) aerial image data. We then built habitat suitability models (Generalized Linear Models—GLMs) to study the effects of landscape metrics and human disturbance on Scaly-sided Merganser presence probability. We found that 1) the Scaly-sided Merganser tended to congregate at river segments with more gravel patches; 2) the Scaly-sided Merganser preferred areas with larger and more contiguous gravel patches; and 3) the number of houses along the river bank (a proxy for anthropogenic disturbance) had significantly negative impacts on the occurrence of the Scaly-sided Merganser. Our results suggest that gravel bars are vital to the Scaly-sided Merganser as shelters from disturbance, as well as sites for feeding and roosting. Therefore, maintaining the exposure of gravel bars in regulated rivers during the low water period in winter might be the key for the conservation of the endangered species. These findings have important implications for understanding behavioral evolution and distribution of the species and for delineating between habitats of different quality for conservation and management.

## Introduction

The river ecosystem plays a fundamental role in global ecosystem services, providing harvestable resources, regulating hydrological regimes and bringing spiritual well-being and recreational opportunities [1]. Rivers are also dispersal corridors and pivotal for biodiversity, therefore often cited as harboring higher abundance of biota [2] or species richness [3]. Composed of a mosaic of aquatic and terrestrial patches, rivers could provide heterogeneous and dynamic habitats for macroinvertebrates, fish and waterbirds. Gravel bars, with braided structure, high relative edge area and connectivity through the landscape, are a characteristic component of river landscapes [4]. These bars are ephemerally inundated at higher flows and subject to wetting and drying processes with changing water levels, thereby alternately offering terrestrial, wet and aquatic habitats [5], and are increasingly, recognized as key sites for many waterbirds [6]. Although the ecological benefits of greater diversity in physical habitat are well known, the influence of spatial distribution of gravel bar mosaics has been less thoroughly studied [5], and the ecological function of gravel bars for waterbirds is not duly appreciated in academic and practitioner communities, ultimately jeopardizing river management and biodiversity conservation.

In this study, we consider the globally endangered Scaly-sided Merganser *Mergus squamatus*, an endemic species restricted to eastern Asia [7] as a case study. Scaly-sided Mergansers are cryptic in nature, and encountered throughout year mostly in small groups (e.g. less than 10 indivaduals). They breed in south-east Russia and northeast China, however, the location of majority of the wintering population is still unknown [8, 9]. Small numbers occur on rivers and fresh water bodies in southern and central China [10–12]. Threats at wintering sites includes dam construction, sand and gravel extraction, industrial and domestic pollution, electrical fishing and anthropogenic disturbance [8]. Scaly-sided Mergansers prefer fast-flowing clear water rivers with riffles, shoals or sand banks in moutainous areas with low levels of human disturbance [8]. Empirical observations [8, 13] are in agreement with such qualitative descriptions of habitat preference of the Scaly-sided Merganser. However, there are few studies quantifying the relationships between the occurrence of Scaly-sided Merganser and gravel bars in riverine landscape, thus bars are usually overlooked in river regulation, and targeted conservation of endangered species is hampered.

We observed the endangered duck persistently preferred some river sections over others within the same river reach in its known wintering distribution [14]. The study is therefore designed to investigate why Scaly-sided Mergansers choose particular habitats, and aims to test the hypotheses that more gravel bars support high occurrence probability of Scaly-sided Merganser, and that occurrence probability is also influenced by human disturbance.

## Materials and Methods

This study was authorized by Department of Forestry of Hunan Province and Yuanshui National Wetland Park.

### Study area

**Fig 1:**
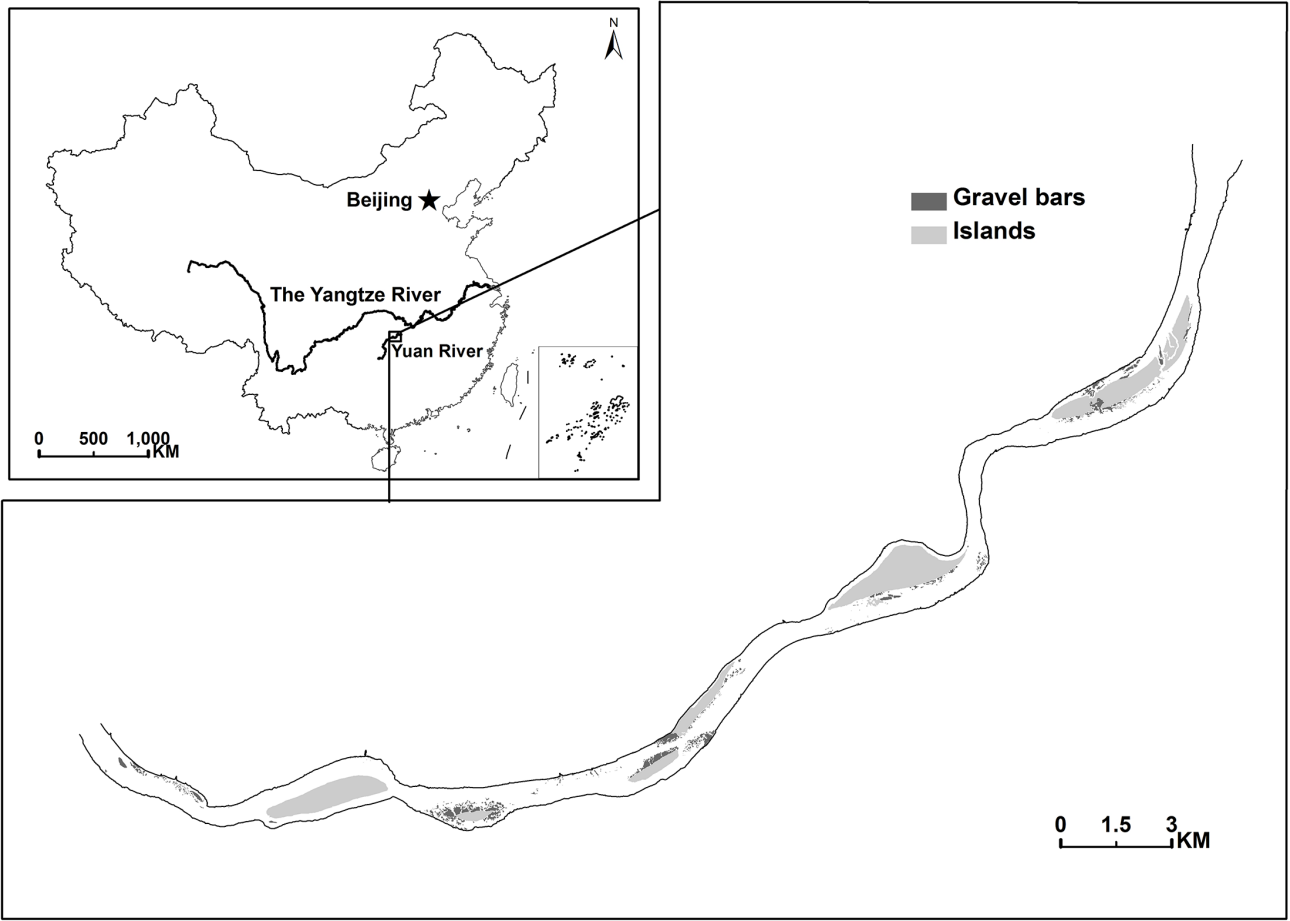
The study area in Yuan River and its location within the Yangtze River Basin in China.

With a length of 1022 km and watershed area of 8.91×10^4^ km^2^, the Yuan River is one of the largest tributaries of the Yangtze River in central China. It originates in Guizhou Plateau, flows through the Wuling Mountain region, and hence brings high water quality and abundant stream fishes. Our study was conducted in a 36 km river section (28°44′-28°52′N, 111°12′-111°30′, Fig 1) in the lower Yuan River. The average water depth is about 6 m and channel width varies from 330 m to 670 m. There are five islands, superior in size and height with most part completely over water surface at high water season, and a range of gravel bars in this river section, with pebbles randomly piled up. Four towns and two villages are distributed along the river banks, which impose different disturbances, including fishing and traffic in river and on riverside roads. This river section supports a population of approximate 40 wintering Scaly-sided Mergansers [14]. The Yuanshui National Wetland Park was established in 2012 specifically for the conservation of this endangered Scaly-sided Merganser population.

### Scaly-sided Merganser occurrence

Field data were collected in three winters (2010/2011, 2011/2012, 2012/2013) when Scaly-sided Mergansers arrived for wintering. Winter is the low water season, and the river is very stable in terms of water level fluctuation. Field survey were conducted every second week from November to February (when the bird population was relatively stable), resulting in a total of 24 surveys.

**Fig 2:**
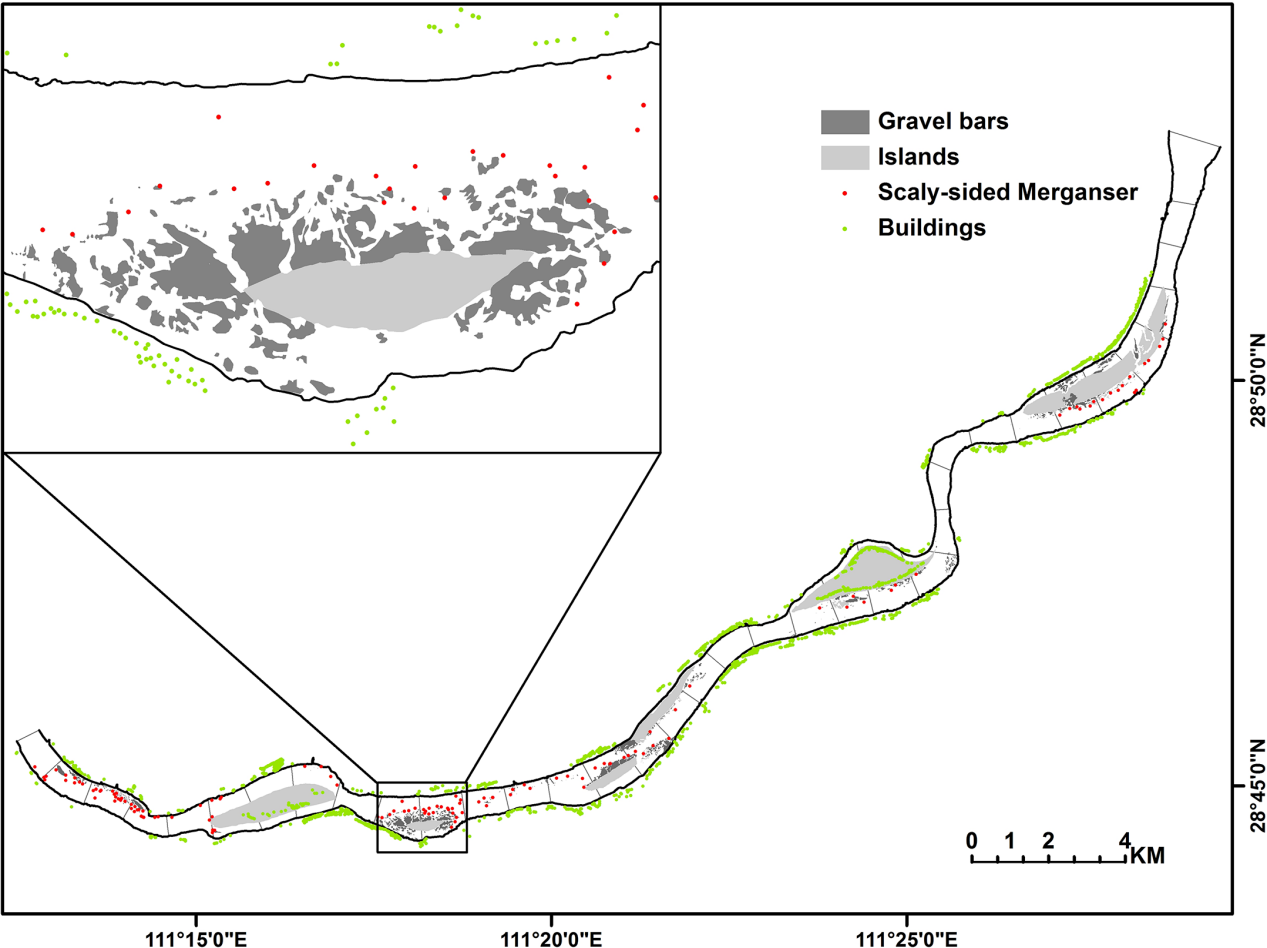
Distribution of Scaly-sided Merganser flocks and gravel bar patches in the 36 km river section of the lower Yuanjiang River. The red dots indicate 127 Scaly-sided Merganser flocks recorded over three winters.Green points represent houses along the river within a 50 m buffer zone. Areas in dark grey are gravel bars in river and area in light grey are islands.

At each survey, we travelled along the 36 km long river course by boat, and used binoculars (8×42) to identify and locate Scaly-sided Merganser, and then mapped the Scaly-sided Mergansers using a GPS (Fig 2). Each survey lasted for four hours (09:00–13:00). During the survey, we kept our boat in the middle of the river to minimize disturbance to birds, and also to ensure that we had a full view of the river section. Distances of less than 10 m between individuals were defined as a flock (i.e. identified as a single occurrence point in mapping). To avoid repeat counting, only birds not flying, e.g. the ducks resting on land or foraging in water were counted. All field surveys were conducted on clear days, avoiding snowy, rainy, or strong windy days, to ensure the quality of the field survey data.

We mapped the presence points from the three winter surveys and summed all points within the 36 predefined 1 km river segments. We termed the ratio of this sum and total points (i.e. 127) as occurrence probability, which is the response variable in our model (Table 1).

**Table 1:** Summary of selected candidate predictor variables and their relevance to Scaly-sided Merganser.

Acronym	Short Description	Median (range)	Relevance
**BLD** *	Number of buildings per km along the river	0.39 (0.02–1.09)	Human disturbance
**NP^**	Number of patches within1 km river length	0.10 (0–0.88)	sites for foraging and roosting
**AREA_AM**	Area-weighted mean patch area	0.16 (0–4.07)	roosting area
**NLSI**	Normalized landscape shape Index	3.37 (0–6.98)	Shape of sites
**SHAPE_AM**	Area-weighted mean Shape Index	1.72 (0–2.89)	Shape of sites
**FRAC_SD**	Standard deviation of fractal dimension	0.04 (0–0.12)	Complexity of sites
**PARA_AM** ^**#**^	Area-weighted mean perimeter area ratio	1.62 (0–6.49)	Complexity of sites
**CONTIG_AM**	Area-weighted mean Contiguity Index	0.94 (0–0.98)	Shape of sites

* divided by 100

^ divided by 10

^#^divided by 1000 to reduce the variability of the predictors for fitted coefficients comparison.

### Gravel patch data

We used the Bing Maps aerial image (2011–12) to derive the gravel patches within the surveyed river section. The images have a spatial resolution of 0.6 meter. We vectorized all the identifiable habitat patches through visual interpretation, and classified them into three types: water, islands, and gravel bars. Islands are much larger and higher, with human habitation or activity, and seldom used by waterfowls. We drew a central line of the river, and equally divided the studied river section into 36 segments by drawing 35 lines perpendicular to the central line (Fig 2). We converted the vector overlays to raster format with 1 m cell size, and used the three patched types for landscape analysis. Buildings along the river banks within the 50 meters buffer zone were depicted as points, and the total number of building within 1 km river segment was used as proxy for the degree of human disturbance (Fig 2). We used ArcGIS 10.4 (ESRI) to prepare all the maps in the study.

### Landscape metrics

All these 36 datasets of the Yuan River were processed in Fragstats 3.3 [15, 16]. According to our aims, we chose patch level metrics to identify gravel bar patches. Many of the landscape metrics are highly correlated [17], especially for metrics in the same class. For the 58 landscape metrics derived from the aerial imagery for our study area, the Pearson’s r ranges from -0.249 (weakly negatively correlated, *standard deviation in patch area* versus *area-weighted mean perimeter area ratio*) to 0.999 (highly identical, many pairs such as *Aggregation Index* vs *Clumpiness Index*). Therefore, pre-screening the variables to address the co-linearity in the following regression is necessary. Furthermore, to avoid over fitting, we limited the predictor variables to three due to the relatively small dataset (i.e. 36 samples). We used a two-step approach to select the candidate variables for model fitting:

1. We grouped the 58 metrics into seven clusters using hierarchical cluster analysis based on dissimilarities defined by the Euclidean distance[18];
2. For the clusters that have two or more members, we checked the Pearson product moment correlation coefficient (Pearson’s r) for the paired variables in each cluster. Not surprisingly, they are highly or strongly correlated (i.e. |r| ≥ 0.70). We selected one variable from each of multi-member clusters by fitting a simple regression model with the occurrence probability, and chose the variable in the model with the highest R^2^ for further investigation (Table 2).

**Table 2:** Summary of GLMs (predictor variables restricted to three or less) supported by survey data.

Model term*	AICc	ΔAICc	AICw	psedu R^2^ ^^^
BLD, CONTIG_AM, and BLD: CONTIG_AM^#^	151.51	0.00	0.33	0.68
BLD, CONTIG_AM, and NP	151.76	0.25	0.29	0.68
CONTIG_AM, NLSI, and PARA_AM	152.35	0.84	0.21	0.67
CONTIG_AM, NP, and PARA_AM	152.79	1.28	0.17	0.67

* See Table 1 for definition of terms

^#^ interaction term

^ Maximum likelihood pseudo *R* ^*2*^

The process reduced the candidate variables to seven: NP, NLSI, AREA_AM, PARA_AM, SHAPE_AM, FRAC_SD, and CONTIG_AM (see Table 1 for explanation of the variables). In addition, we calculated the building density along the river banks (buildings per 100 m river length) and used it as a proxy for human disturbance intensity. A summary of the candidate variables was listed in Table 1.

### Habitat modeling

Generalized linear models (GLMs) are commonly used for modeling habitat suitability because of their strong statistical foundation and ability to realistically model ecological relationships [19]. To specify an appropriate link function in GLM, we fitted four probability distribution functions (PDF) of the dependent variable (i.e. occurrence probability), namely Poisson, negative binomial, zero-inflated Poisson and Gaussian, and used Akaike's information criterion (AIC) to select the best PDF. As negative binomial describes the distribution best, we used log link and negative binomial error distribution for our habitat model building.

We first developed a full GLM model that includes all eight selected predictor variables (Table 1) and the interactions between BLD and other landscape metrics using the “MASS” package [20] within the R Language and Environment for Statistical Computing, version 3.0.1[21]. We included the interaction terms in the modelling to explore the “shielding” effect of gravel bars on the Scaly-sided Merganser from human disturbances. We then used the backward stepwise technique to select the best fitted GLM which resulted in six predictor variables. Due to the relative small sample size, it is necessary to restrict the number of terms to three to avoid over-fitting [20, 22]. We also diagnosed the models for multicollinearity by calculating the condition indexes [23] using the package "perturb" [24]. We used the automated model selection function “dredge” in the “MuMIn” package[25] to limit the maximum term to three and reported the model with lowest AIC_c_ (small sample corrected AIC) as the final model. We also assessed the relative likelihood of each candidate model by AIC weight (AIC_w_). In order to evaluate the relative importance of the predictive variables for the occurrence of the Scaly-sided Merganser, we followed Burnham and Anderson [22] to sum the AIC_w_ over all models with ΔAIC_c_ less than 4. To address the uncertainty in the estimated coefficients of predictor variables caused by small sample size, we re-fitted the final model using the Markov Chain Monte Carlo (MCMC) approach implemented in the MCMC package [26]. We simulated a sample of 55,000 iterations from the posterior distribution using standard Gibbs sampling method, and calculated the 95% intervals of the coefficients after 50,000 burn-in and a thinning interval of 1000.

## Results

During the whole 24 surveys (2011–2013), we counted 514 Scaly-sided Mergansers, belonging to 127 flocks in total (Fig 2). The number of individual in a flock varied from 1 to 20.

**Fig 3:**
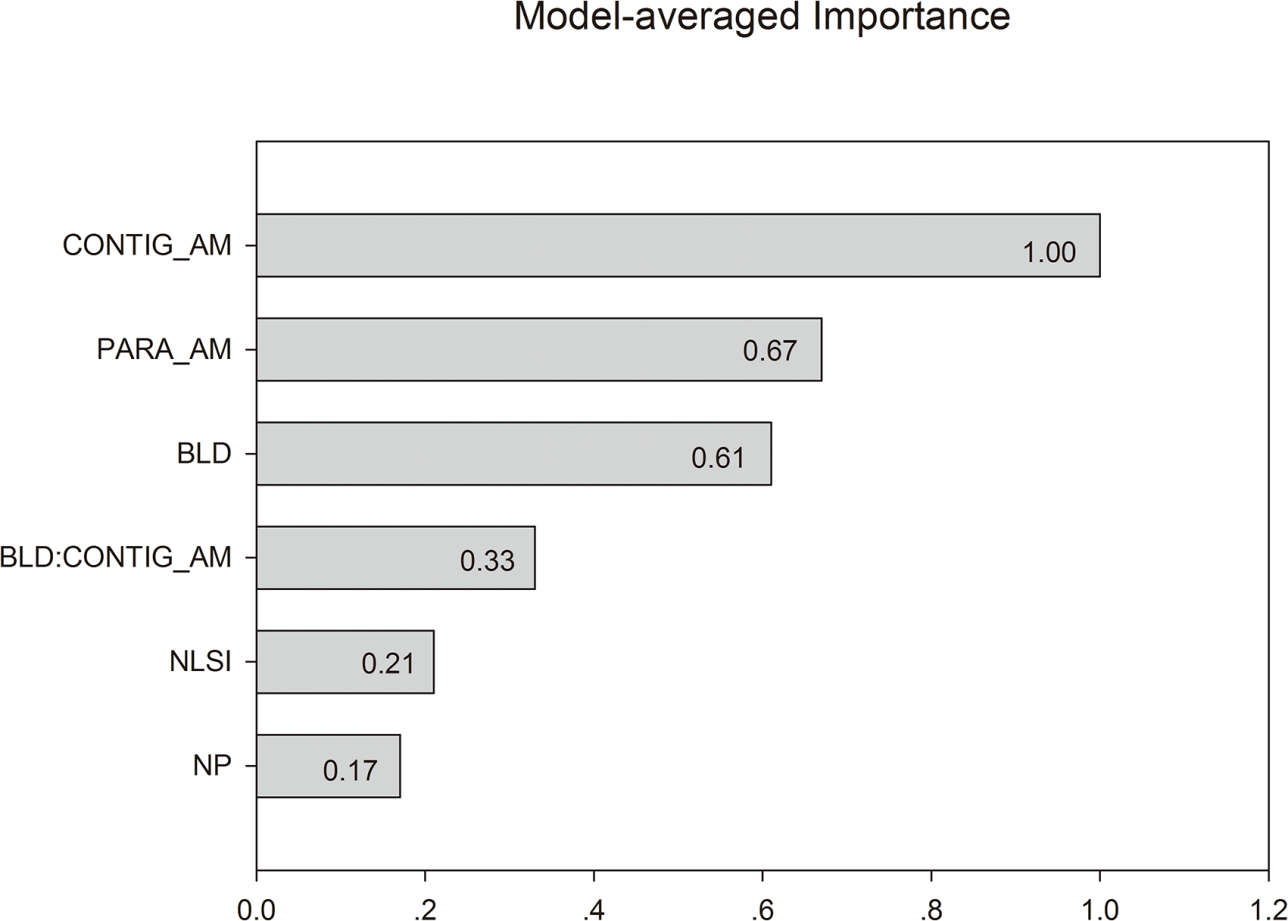
The importance of predictor variables to Scaly-sided Merganser occurrence based on the sum of AIC_w_ over all models listed in Table 2.

There are four feasible GLMs for the Scaly-sided distribution data based on model AIC_c_ (ΔAIC_c_ <2, Table 2). All these models had considerable prediction power: based on the psedu-*R* ^*2*^ the models explained 67% -78% of the variations in the duck occurrence data (Table 2). In addition, the largest condition number was 4.35 for Model 3 in Table 3), suggesting there is no mulitcollinearity problem [23]. Collectively, these models involved five variables: BLD, CONTIG_AM, PARA_AM, NLSI, NP, and the interaction between BLD and CONTIG_AM. Among these predictor variables, CONTIG_AM was the top variable with the highest importance to predict the occurrences of the Scaly-sided Merganser based on model AIC_w_. PARA_AM and BLD were comparably important, and NLSI and NP were the two least important variables (Fig 3). Furthermore, the BLD/CONTIG_AM interaction term also had considerable importance (Fig 3).

**Table 3:** Coefficients of the top three GLM and the creditable interval based on Markov Chain Monte Carlo sampling.

Models		GLM			Bayesian Inference		
		Estimate	Std. Error	p value	Lower 2.5%	Medium	Upper 97.5%
**Model 1**	BLD	-5.35	1.99	**	-5.41	-1.75	2.11
	CONTIG_AM	1.09	1.19	NS	1.11	4.11	7.01
	BLD:CONTIG_AM	5.21	1.67	**	-5.83	-1.44	3.23
**Model 2**	BLD	-2.02	0.72	**	-5.95	-2.88	0.75
	CONTIG_AM	4.11	0.90	***	2.35	5.58	8.52
	PARA_AM	-0.57	0.19	**	-2.01	-1.08	-0.03
**Model 3**	CONTIG_AM	3.55	0.96	***	1.34	4.66	8.34
	NLSI	0.87	0.39	*	-0.94	0.42	1.72
	PARA_AM	-2.12	0.81	*	-3.05	-1.40	0.35
**Model 4**	CONTIG_AM	3.26	0.97	*	1.41	4.21	7.49
	NP	1.85	0.70	*	0.71	4.83	9.10
	PARA_AM	-0.50	0.22	*	-1.81	-0.88	0.04

Significant: p > 0.1, NS

*p < 0.1

**p<0.05

*** p<0.01

The model with BLD, CONTIG_AM and their interaction tern as predictor variables had the lowest AIC_c_, and was ranked as the best model supported by data. However, the ΔAIC_c_ of four possible models were less than 2, we considered them were equally supported. Therefore, we performed Bayesian inference via MCMC for the four models and calculated the creditable intervals of the coefficients (Table 3).

**Fig 4:**
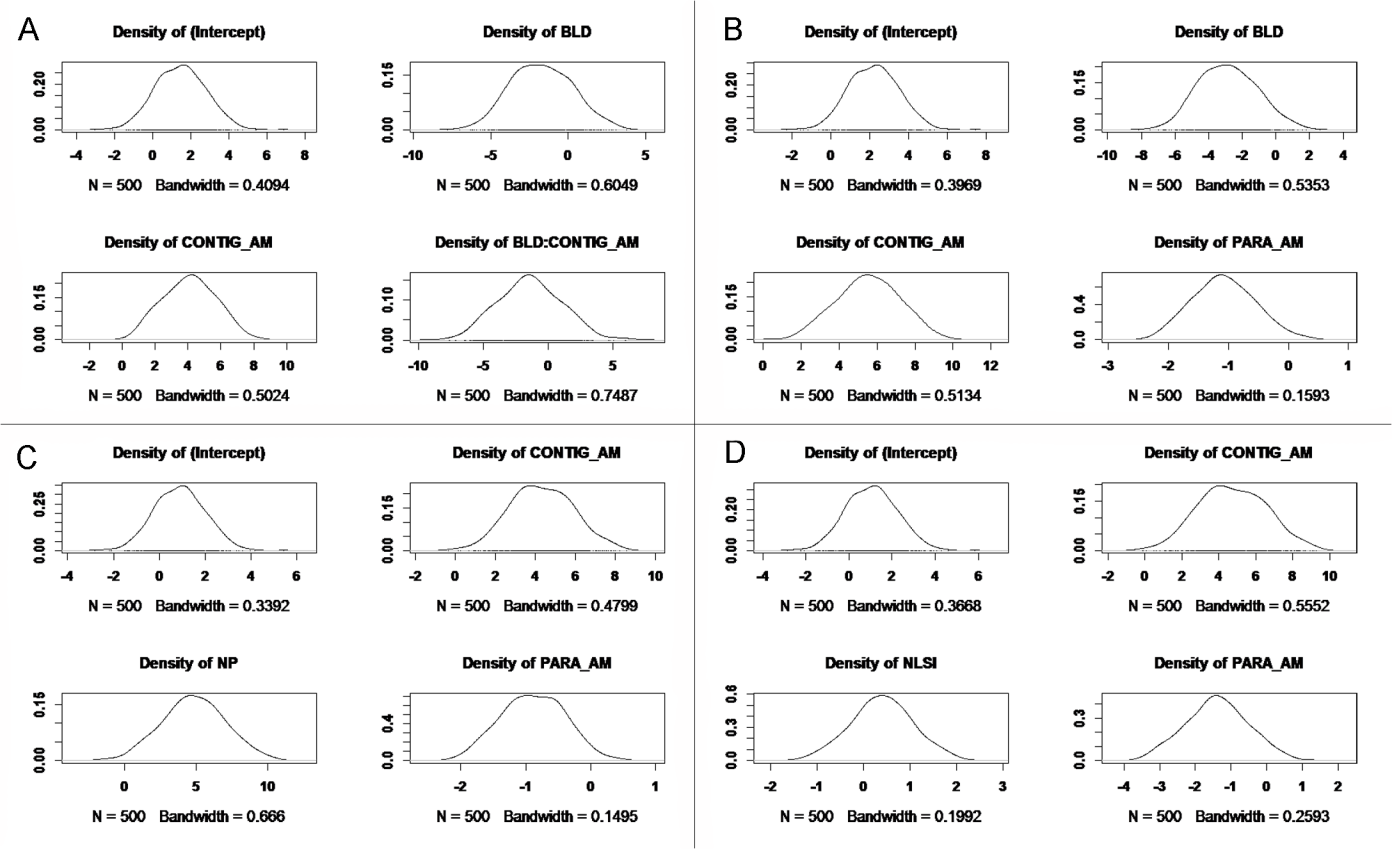
The density of estimated coefficients for A) Model 1, B) Model 2 C) Model 3, and D) Model 4 through 50,000 iterations of Gibbs sampling.

The occurrence of the Scaly-sided Merganser was significantly negatively affected by BLD, and this was consistent for both the GLM estimates (Table 3) and the Bayesian inferred posterior density distribution (Table 3 and Fig 4B). In addition, the GLM estimation of -2.02 was close the medium of -2.88 from MCMC posterior sampling for the model which included BLD as main factor only (i.e. model 2).

The occurrence of the Scaly-sided Merganser had significantly positive relationship with CONTIG_AM. The significantly positive relationship was consistence across all the plausible GLMs although their estimation varied (i.e. the coefficients are 1.09, 4.11, 3.55, and 3.26 for models 1, 2, 3 and 4 respectively, Table 3). The significant level of positive relationship between Scaly-sided Merganser presence and CONTIG_AM was verified by Bayesian inference (i.e. the 95% intervals were all positive and 0 was not included, Table 3 and Fig 4). The MCMC estimates were consistently higher across the models (Table 3).

Based on the fitted GLMs, PARA_AM negatively affects the occurrence of the Scaly-sided Merganser although the coefficient in model 2 and model 4 was considerably higher than in model 3. Furthermore, the Bayesian estimates were higher for models 2 and 4, but lower for model 3. In addition, the 95% intervals included 0 for model 3 and 4 (Table 3) although the distribution density was heavily skewed to the left (negative) (Fig 4).

The posterior MCMC showed a strong positive relationship between NP and the occurrence of the Scaly-sided Merganser (Fig 4D and Table 3). The coefficient was 1.85 and 4.83 based on GLM and Bayesian estimation, respectively. For NLSI, although the GLM indicated a significantly positive effect, the Bayesian posterior inference was not conclusive (Table 3): the 95% internals were -0.94 and 1.72, and the distribution density was slightly skewed to the positive range (Fig 4C).

## Discussion

### Landscape metrics

Our results indicated that the occurrence of the Scaly-sided Merganser had significantly negative relationship with the perimeter-area ratio and positive with the contiguity index. Perimeter-area ratio reflects both the area and shape of a patch. Large patches tend to have more irregular shapes, thus landscapes with larger patches could represent higher values for area-weighting patch shape indexes [27]. This may lead them to be more related to patch size than to patch shape [28]. In agreement with our findings, Schlossberg and King [29] also concluded that small and irregularly-shaped and clearcut patches were less likely to provide quality habitat for shrubland species. Similarly, Helzer and Jelinski [30] found that the probability of occurrence for six common grassland avian species was significantly inversely correlated with perimeter-area ratio. Aligning with these findings, Embling et al. [31] proposed a methodology combining spatial modelling with perimeter-to-area ratio for selecting protected areas of harbor porpoises, as well as other animal species. The contiguity index is a metric that assesses the spatial connectedness of cells within a grid-cell patch to provide an index of patch boundary configuration and thus patch shape [15]. Therefore, patches that are large and contiguous have larger Contiguity Index values [15]. The positive relationship between the occurrence of the Scaly-sided Merganser and Contiguity Index suggested that the species preferred areas with larger and more contiguous gravel patches that may serve as shelters from disturbance, as well as sites for foraging and roosting.

### Gravel bar’s function

Larger gravel bars could improve the function of river section as possible roosting sites. The incessant, strongly turbulent flow requires strong rooting to resist, or significant expenditure of energy to withstand [32]. Scaly-sided Merganser uses gravel bars to roost and preen, which could minimize the necessary expenditure of energy to maintain position for other activities such as feeding and social interaction. These resting and maintenance behaviors could account for 39% and 24% of time budget in male and female during days [13].

The composition and configuration of the river landscape determines the fish assemblage diversity and abundance [33, 34]. As in-stream refugia for feeding and spawning, larger gravel bars also harbor a diversity of organisms, and increase the abundance and diversity of fish and macroinvertebrates, providing rich food sources for waterfowls. Many species (e.g. darter, salmon) favor riffles and runs with substrates of gravel and sand. For lithophilous species, the bar edges offers both shelter and proximity to spatially segregated food resources [35]. Indeed, compared with the nearby unvegetated sediment areas, the abundance of predatory fishes associated with cobble and boulder patches was significantly higher, and within cobble and boulder patches, abundances were significantly higher along the edge than in the interior [36]. Furthermore, proximity to cobble patch edges also influences crustacean distribution [37]. The Scaly-sided Mergansers have a wide range of diets, mainly stream fish, supplemented with benthic invertebrates, aquatic larvae, frogs, etc [38, 39]. They search for prey with their eyes below the water surface or dive into the water column. Synchronous diving and drifting the fish assemblage to the bar is a strategy in group foraging of Scaly-sided Merganser. Although several studies indicated that mean food intake rate (MFIR) increased in groups [40], group hunting and foraging were generally assumed to be a strategy to decrease predation risk and led to increased food competition [41, 42]. Gravel bars are therefore especially important for such “group effects”.

In addition, large bars might promote visual isolation, which is thought to affect the territory size of visually oriented animals. Some scholars [43] suggested that visual isolation reduced intraspecific competition and increased breeding waterfowl pair density in Manitoba, Canada. Furthermore, large gravel bars interspersing with currents may not only reduce the inter- and intra- specific confrontations, but also minimize human disturbances both in stream and from river banks. Anthropogenic disturbance, represented as the number of houses along the river bank in our study, had significantly negative impacts on the occurrence of the Scaly-sided Merganser. This is in agreement with anecdotal records that most wintering sites for Scaly-sided Merganser were away from major centers of human habitation [8]. It is well documented that disturbance from human activities can be energetically costly due to decreased foraging efficiency and increased escape activities [44], which could cause not only temporary changes in behavior but also locally affect the temporal and spatial distribution of migratory and wintering waterfowl [45]. For example, fishing and boat traffic may increase alertness and even escape activities in pelicans and coots [46]. The "best" fitted model (Model 1 in Table 3) exhibited an interaction effect of human disturbance and gravel bars on the occurrences of Scaly-sided Merganser. The opposite effect of the BLD and the interaction (BLD: PARA_AM) clearly revealed the bar’s function as shelter or fence from outer disturbance. With the presence of human activities, birds on the protected side of the fence or barrier responded similarly to birds at the low visitation control site [47]. In the highly populated south and central China, rivers with low human activities are extremely rare. Scaly-sided mergansers could use large gravel bars to keep themselves safe behind the “cordon”, which acts a buffer before their flight initiation.

### Conservation implications

One of the most serious threat associated with wintering Scaly-sided Merganser is dam construction [8]. Released clear water from dams scours downstream bars, reducing bar number and area [48, 49] and reducing the embeddedness of the river bottom substrate that benefit macroinvertebrate and fish production [50]. Maintaining the exposure of gravel bars in regulated rivers during winter is critical for this endangered species, and should be take in to consideration at the very beginning in municipal planning and dam construction. Moreover, the electricity hydropower plants could adjust the operational schedule, e.g. to run in the night or early morning, and keep low water level during day as long as possible. Equally important, within-channel alluvial gravel extraction is one of the important forms of anthropogenically induced morphological change in river channels [51], and is causing gravel-size sediment starvation [52], which leads to lowering of riverbed and decrease of gravel bars, therefore sand mining and gravel extraction should be restricted and regulated.

## Conclusion

In the research, we found that 1) the Scaly-sided Merganser tended to congregate at river reaches with more gravel patches; 2) the Scaly-sided Merganser preferred areas with larger and more contiguous gravel patches; and 3) the number of houses along the river bank (a proxy for anthropogenic disturbance) had significantly negative impacts on the occurrence of the Scaly-sided Merganser. Our results suggested that gravel bars are vital to the Scaly-sided Merganser as shelters from disturbance, as well as sites for feeding and roosting. Therefore, maintaining the exposure of gravel bars in regulated rivers during the low water period in winter might be the key for the conservation of the endangered species. The findings provide a quantitative association between wintering habitat of Scaly-sided Merganser and geomorphic features, as well as human disturbance, an association that previous research has documented in only qualitative terms, therefore have important implications for understanding behavioral evolution and distribution of the species and for delineating between habitats of different quality for conservation and management.
